# Micro Magnetic Field Sensors Manufactured Using a Standard 0.18-μm CMOS Process

**DOI:** 10.3390/mi9080393

**Published:** 2018-08-07

**Authors:** Yen-Nan Lin, Ching-Liang Dai

**Affiliations:** Department of Mechanical Engineering, National Chung Hsing University, Taichung 402, Taiwan; g102061073@mail.nchu.edu.tw

**Keywords:** micro sensor, Hall effect, magnetic field, magnetotransistor

## Abstract

Micro magnetic field (MMF) sensors developed employing complementary metal oxide semiconductor (CMOS) technology are investigated. The MMF sensors, which are a three-axis sensing type, include a magnetotransistor and four Hall elements. The magnetotransistor is utilized to detect the magnetic field (MF) in the x-axis and y-axis, and four Hall elements are used to sense MF in the z-axis. In addition to emitter, bases and collectors, additional collectors are added to the magnetotransistor. The additional collectors enhance bias current and carrier number, so that the sensor sensitivity is enlarged. The MMF sensor fabrication is easy because it does not require post-CMOS processing. Experiments depict that the MMF sensor sensitivity is 0.69 V/T in the x-axis MF and its sensitivity is 0.55 V/T in the y-axis MF.

## 1. Introduction

Magnetic field (MF) sensors, which are important components, are applied in industrial apparatuses, automation equipment, cable-stayed bridges, electrical devices and portable electronic instruments [1,2,3,4]. Traditional MF sensors [5,6], which were not manufactured by microfabrication, were not only large volume, but also high cost. The advantages of micro magnetic field (MMF) sensors are small volume and low cost. Micro-electro-mechanical-system (MEMS) technology could be utilized to fabricate micro sensors [7,8,9,10,11]. Several MMF sensors were manufactured using MEMS technology. For instance, Mian [12] developed resonant MMF sensors fabricated by the surface micromachining process. The sensor structure contained microbeams and a membrane, the material of which was a stack of double polysilicon layers. Based on the Lorentz force principle, the MMF caused a capacitance change upon sensing an MF. The Lorentz force resonant MEMS magnetic field sensors were proposed by Park [13]. The MMF sensors had a micromirror actuated by the Lorentz force that was generated using a sinusoidal current and an incident MF. The rotation angle of the micromirror was recorded using an optical measurement. Dennis [14] used a CMOS process to manufacture resonant MMF sensors. The sensor was fabricated using the stacked layers of the CMOS process, and a post-CMOS processing with reactive ion etch (RIE) dry etching was adopted, releasing the device structure. The sensor shuttle was excited using the Lorentz force and external MF, and the resonance amplitude was detected by an optical instrument. These resonant MMF sensors [12,13,14] required movable suspension structures, so sacrificial layer technology was used to release the suspension structures. For example, after completion of the CMOS process, the resonant MMF sensors, proposed by Dennis [14], used an RIE dry etching post-processing to obtain the suspension structures of the devices. In this work, we design a magnetotransistor/Hall element MMF sensor without a suspension structure using a CMOS process, so the sensor does not need post-CMOS processing. Therefore, the sensor fabrication in this work is easier than that of these sensors [12,13,14].

A one-axis magnetotransistor MMF sensor, presented by Tseng [15], was fabricated by a standard 0.18-μm CMOS process of Taiwan Semiconductor Manufacturing Company (TSMC). The MMF sensor had a sensitivity of 354 mV/T. Furthermore, Tseng [16] adopted the same method to design a three-axis magnetotransistor MMF sensor that was also made using a standard 0.18-μm CMOS process of TSMC. The MMF sensor had a sensitivity of 6.5 mV/T in the x-axis and a sensitivity of 0.4 mV/T in the y-axis. A two-dimensional Hall MMF sensor with a lateral magnetotransistor and magnetoresistor, developed by Yu [17], was produced using a standard 0.35-μm CMOS process. The sensitivity of the MMF sensor was 0.385 V/(A·T) at a bias current of 100 mA. Sung [18] proposed a two-dimensional Hall MMF sensor manufactured utilizing a standard 0.35-μm CMOS process. The MMF sensor was composed of a bulk magnetotransistor, a vertical magnetoresistor and a vertical magnetotransistor. The sensitivity of the sensor was 1.92 V/(A·T) at a bias current of 20 mA. With the same design method, Sung [19] developed a three-dimensional Hall MMF sensor with a bandgap reference and readout circuit made using a standard 0.18-μm CMOS process. The MMF sensor contained one-dimensional lateral Hall sensor and a two-dimensional vertical Hall sensor. The MMF sensor had a sensitivity of 5943 V/(A·T) at a bias current of 6.25 mA in the x- and y-axis MF and a sensitivity of 14,790 V/(A·T) at a bias current of 6.25 mA in the z-axis MF. The Hall MMF sensors, proposed by Xu [20], were fabricated by the 0.18-μm high voltage (HV) CMOS process for sensing low MF. The sensors consisted of a Hall plate with a switching cross-shape. Zhao [21] utilized a CMOS process to make nano-polysilicon transistor MMF sensors. A nano-polysilicon/single silicon junction was adopted as a sensing layer. The nano-polysilicon transistors were fabricated on silicon substrate with high resistivity. The two-dimensional MMF sensors, develop by Yang [22], included four magnetic transistors. The MMF sensors were manufactured on a high resistivity silicon wafer using microfabrication technology, and they were packaged on printed circuit boards. The sensor sensitivity in the x-axis was 366 mV/T, and its sensitivity in the y-axis was 365 mV/T, respectively. These micro sensors [15,17,20,21,22] manufactured using CMOS technology were one-axis and two-axis MMF sensors. Therefore, three-axis MMF sensors in this work are fabricated using a standard 0.18-μm CMOS process of TSMC, and the sensitivity of the sensors is higher than that of Tseng [16].

Various MEMS actuators and sensors, which are manufactured utilizing a CMOS process, are called CMOS-MEMS devices [23,24,25,26,27]. We adopt CMOS-MEMS technology to develop three-axis MMF sensors. MMF sensors are composed of a magnetotransistor and four Hall elements. The magnetotransistor is designed to detect MF in the x-axis and y-axis. Four Hall elements are designed to sense MF in the z-axis. These CMOS-MEMS magnetic field sensors [28,29,30] needed a post-CMOS processing [31] to form suspension structures. The fabrication of the MMF sensors in this study is consistent with the CMOS process and does not need post-CMOS processing.

## 2. Structure of MMF Sensor 

**Figure 1:**
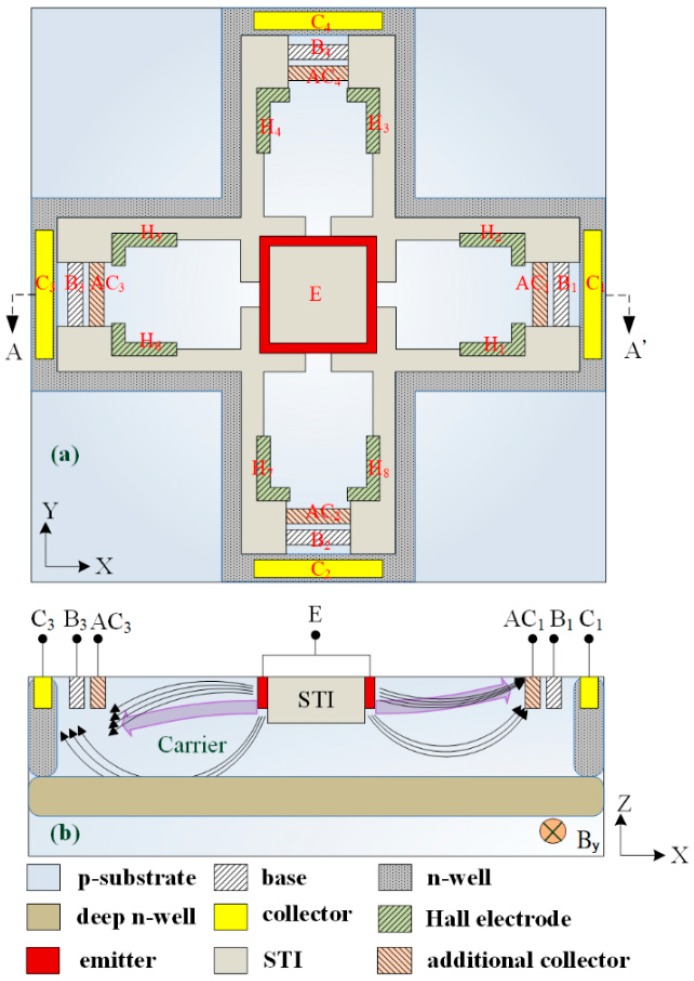
Micro magnetic field (MMF) sensor: (**a**) schematic structure; (**b**) cross-sectional view along line AA’. STI, shallow trench isolation; E, emitter.

Figure 1a demonstrates the MMF sensor structure, where E denotes the emitter, B_1_, B_2_, B_3_ and B_4_ are the bases, C_1_, C_2_, C_3_ and C_4_ are the collectors, AC_1_, AC_2_, AC_3_ and AC_4_ are the additional collectors and H_1_, H_2_, H_3_, H_4_, H_5_, H_6_, H_7_ and H_8_ are the electrodes of the Hall elements. 

**Figure 2:**
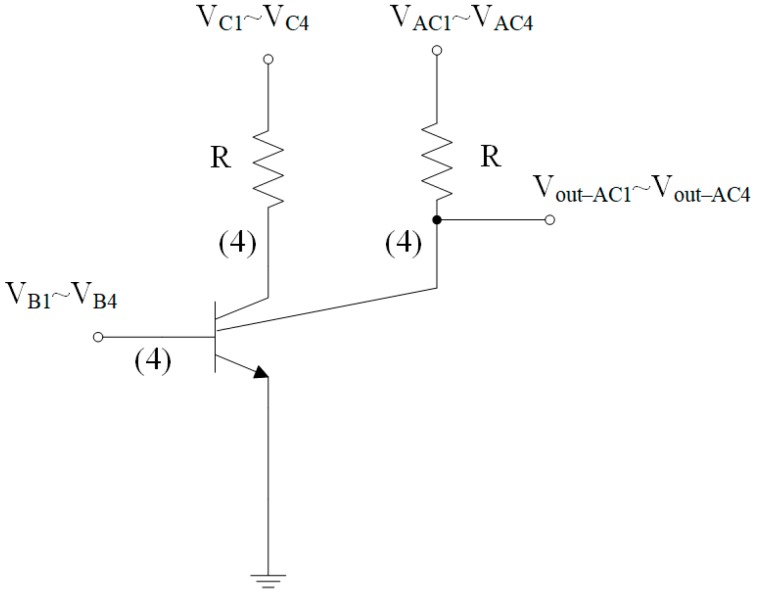
Equivalent circuit for the magnetotransistor. V, bias voltage; C, collector; R, resistor; AC, additional collector; B, base.

The MMF sensor includes a magnetotransistor and four Hall elements. The magnetotransistor is employed to detect MF in the x-axis and y-axis, and the four hall elements are utilized to sense MF in the z-axis. In addition to the emitter, bases and collectors, the additional collectors are introduced into the magnetotransistor. The additional collectors can increase bias current, so that the emitter induces more electron carriers. Shallow trench isolation (STI) oxide is used to confine the current direction, so that leakage current reduces. Figure 2 illustrates the equivalent circuit for the magnetotransistor, where R represents the resister, V_C1_, V_C2_, V_C3_ and V_C4_ are the bias voltage of the collectors, V_AC1_, V_AC2_, V_AC3_ and V_AC4_ are the bias voltage of the additional collectors, V_B1_, V_B2_, V_B3_, and V_B4_ are the bias voltage of the bases, V_out-AC1_, V_out-AC2_, V_out-AC3_ and V_out-AC4_ are the output voltages and the symbol (4) denotes the corresponding circuit repeated four times.

Figure 1b demonstrates a cross-sectional view of the MMF sensor. The sensing mechanism of the magnetotransistor is explained as follows. As shown in Figure 1b, when the bias voltages are applied to the collectors, the bases and the additional collectors, carriers produce a movement from the emitter to the additional collectors AC_1_/AC_3_, the bases B_1_/B_3_ and the collectors C_1_/C_3_. Given a magnetic field in the y-axis, carriers (on the right in Figure 1b) are bent upward because of the action of the Lorentz force. The carriers have difficulty passing across the additional collector AC_1_ to the base B_1_ and collector C_1_. Most of carriers move to the additional collector AC_1_, resulting in the current increment of the additional collector AC_1_. At the same time, carriers (on the left in Figure 1b) are bent downward owing to the action of the Lorentz force. Most of the carriers move across the additional collector AC_3_ to the base B_3_ and collector C_3_, leading to the current decrement of the additional collector AC_3_. Therefore, this action produces a voltage difference between the additional collectors AC_1_ and AC_3_ in the y-direction MF. As shown in Figure 2, the additional collectors AC_1_ and AC_3_ respectively connect to a resistor R, so the voltage difference of the additional collectors AC_1_ and AC_3_ is obtained by V_out-AC1_ − V_out-AC3_, which is the sensor output voltage in the y-axis MF. Similarly, when the bias voltages are applied to the collectors, the bases and the additional collectors, carriers produce a movement from the emitter to the additional collectors AC_2_/AC_4_, the bases B_2_/B_4_ and the collectors C_2_/C_4_. Given a magnetic field in the x-axis, carriers that move to the additional collector AC_2_ are bent downward by Lorentz force, leading to the current decrement of the additional collector AC_2_. Additionally, carriers that move to the additional collector AC_4_ are bent upward by the Lorentz force, resulting in the current increment of the additional collector AC_4_. The current between both additional collectors AC_2_ and AC_4_ generates an imbalance, so the additional collectors AC_2_ and AC_4_ produce a voltage difference. As shown in Figure 2, the voltage difference of the additional collectors AC_2_ and AC_4_ can be obtained by V_out-AC4_ − V_out-AC2_, which is the sensor output voltage in the x-axis MF.

**Figure 3:**
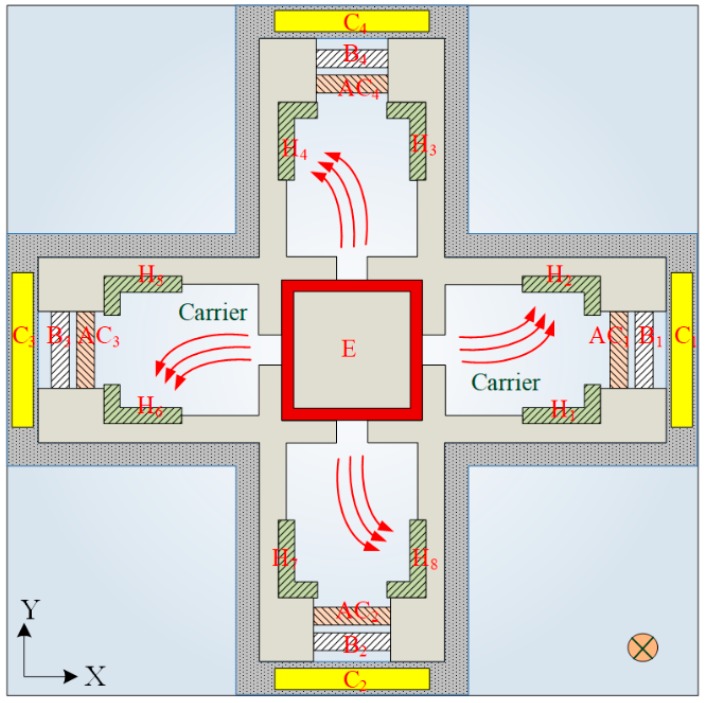
Carrier path in the MMF sensor under z-direction MF. H, Hall electrode, E, emitter.

The MMF sensor has four Hall elements used to detect z-direction MF. Figure 3 presents the carriers’ path in the MMF sensor under z-direction MF. As shown in Figure 3, when the bias voltages are applied to the collectors, bases and additional collectors, carriers cause a movement from the emitter to the additional collectors AC_1_, AC_2_, AC_3_ and AC_4_. Given an MF in the z-axis, carriers are bent toward the Hall electrodes H_1_, H_3_, H_5_ and H_7_ by the Lorentz force. The current causes an imbalance between the electrodes H_1_ and H_2_, leading to the generation of a Hall voltage between the electrodes H_1_ and H_2_. Similarly, the Hall voltages between the electrodes H_3_/H_4_, H_5_/H_6_ and H_7_/H_8_, respectively, are generated in z-direction MF. The Hall voltages in series are the sensor output voltages in z-axis MF.

The Sentaurus TCAD, which is a finite element method software, was utilized to simulate the MMF sensor performance. According to the structure in Figure 1a, the model of the MMF sensor was constructed. To save computation time, one-quarter of the MMF model was established because the MMF sensor structure was symmetric. Then the method of Delaunay triangulation was employed to mesh the MMF model. The approach of the Poisson electron hole was used to compute the coupling effect of MF and the electrical field, and the method of Bank/Rose was utilized to solve the carrier density distribution of the MMF sensor.

**Figure 4:**
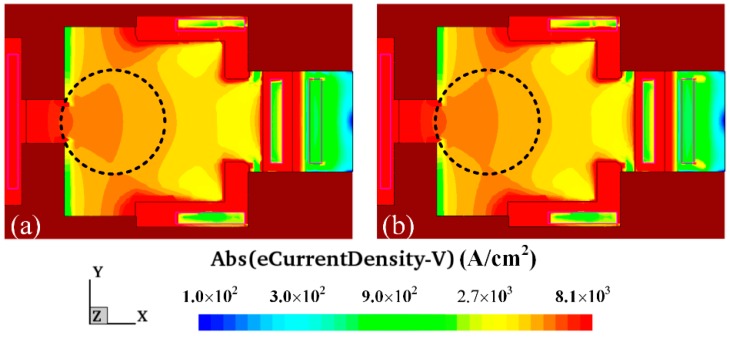
Carrier density distribution of the MMF sensor under the y-direction magnetic field.

In this simulation, bias voltages of 1.5 V, 4.5 V and 4.5 V were supplied to bases, collectors and additional collectors, respectively. A magnetic field of 250 mT was given in the y-axis. Figure 4 shows the simulated carrier density distribution of the MMF sensor under the y-direction magnetic field. Figure 4a illustrates the carrier density distribution of the MMF sensor without a magnetic field. Figure 4b reveals the carrier density distribution of the MMF sensor with a magnetic field of 250 mT in the y-axis. By the comparison of the simulated results in Figure 4a,b, the current density of the path from the emitter to the additional collector increases.

**Figure 5:**
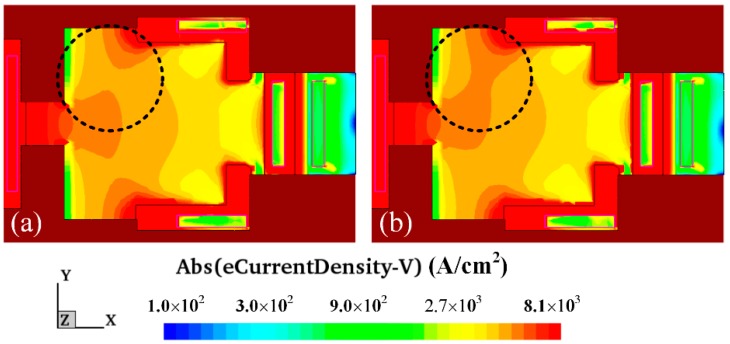
Carrier density distribution of the MMF sensor under the z-direction magnetic field.

The carrier density distribution of the MMF sensor in the z-direction magnetic field was computed with the same simulation approach. In this computation, bias voltages of 1.5 V, 4.5 V and 4.5 V were supplied to bases, collectors and additional collectors, respectively. A magnetic field of 250 mT was applied in the z-axis. Figure 5 shows the simulated carrier density distribution of the MMF sensor under the z-direction magnetic field. Figure 5a demonstrates the carrier density distribution of the MMF sensor without a magnetic field. Figure 5b presents the surface carrier density distribution of the MMF sensor with a magnetic field of 200 mT in the z-axis. As illustrated in Figure 5a,b, carriers are bent toward the top Hall electrode.

## 3. Fabrication of MMF Sensor

**Figure 6:**
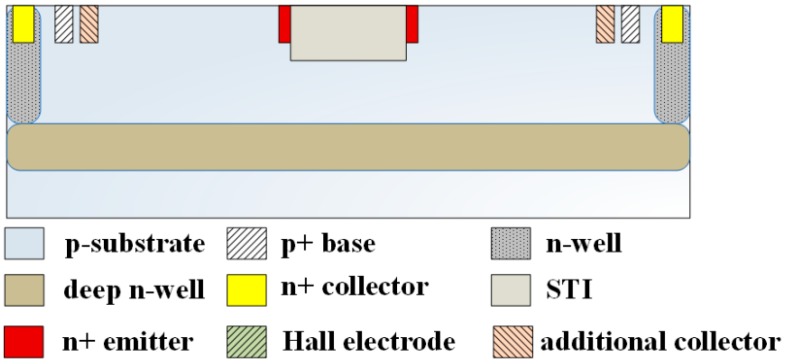
Cross-sectional view of the MMF sensor after the CMOS process.

**Figure 7:**
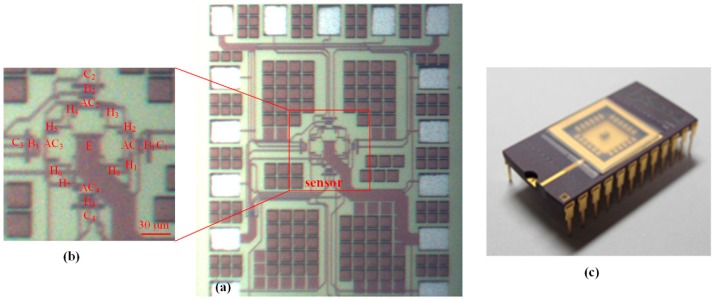
Picture of the MMF sensor chip.

The layout of the MMF sensor was designed in accordance with the structure in Figure 1a. The TSMC used a 0.18-μm CMOS process to manufacture the MMF sensor according to the MMF sensor layout. Figure 6 demonstrates the cross-sectional structure of the MMF sensor after completion of the CMOS process. As demonstrated in Figure 6, the MMF sensor was fabricated on p-type substrate. The MMF sensor consisted of an emitter, four collectors, four bases, four additional collectors and eight Hall electrodes. The emitter was n-type silicon doping phosphorus. The collectors and the additional collectors were n-type silicon doping phosphorus, and the bases were p-type silicon doping boron. Hall electrodes were n-type silicon doping boron. The deep n-well layer, which was a buried layer, was connected to n-well layer to confine the current downward moving range and to reduce leakage current. The STI oxide, which surrounded the emitter edge to confine the current moving direction, would reduce leakage current. An image of the MMF sensor is presented in Figure 7a. Figure 7b shows the magnified picture of the sensor with a scale bar. Fabrications of the other MEMS MMF sensors [12,13,14] were more complicated than that of the MMF sensor because the MMF sensor did not require any post-processing. The MMF sensor chip was wire-bonded and packaged on a frame. Figure 7c shows the MMF sensor picture after packaging.

## 4. Results

**Figure 8:**
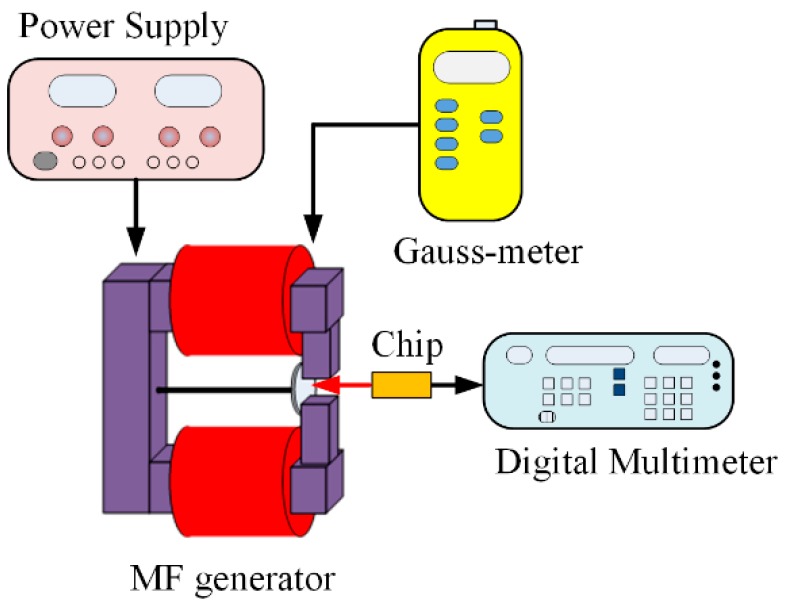
Experiment setup for the MMF sensor.

A magnetic testing system was employed to measure the MMF sensor performance. Figure 8 demonstrates a magnetic testing system [15], and the system includes a Gauss-meter (GM08-1029, Hirst, Falmouth, U.K.), an MF generator (developed by our lab), a power supply (GPC-3030DQ, Gwinstek, New Taipei City, Taiwan) and a digital multimeter (34405A, Agilent, Santa Clara, CA, USA). The magnetic generator was employed to generate an MF to test the MMF sensor. The power supply was used to provide power to the MF generator. The Gauss-meter was used to test the magnetic magnitude excited by the MF generator. The digital multimeter was utilized to record the MMF sensor output voltage.

**Figure 9:**
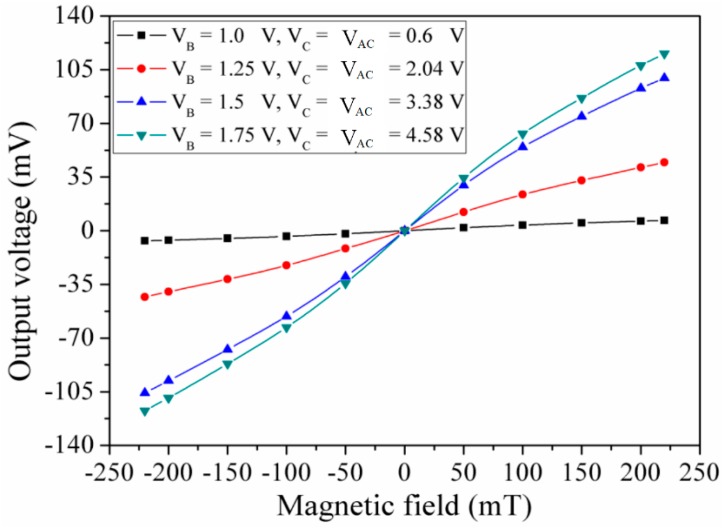
Measured output voltage in the y-direction MF.

The MMF sensor was composed of a magnetotransistor and four Hall elements. The magnetotransistor was used to detect MF in the x- and y-directions, and the Hall elements were utilized to measure the magnetic field in the z-direction. First, the MMF sensor performance in the x-direction MF was tested. The MMF sensor was set in the magnetic testing system. An MF range of −220–220 mT generated by the MF generator was supplied to the MMF sensor, and the MF magnitude was calibrated using the Gauss-meter. Bias voltages were applied to the bases, collectors and additional collectors. An MF in the y-direction was applied to the MMF sensor. The digital multimeter measured the voltage difference of the additional collectors AC_1_/AC_3_ in the MMF sensor. Figure 9 depicts the measured output voltage of the MMF sensor in the y-direction MF. When V_B_ = 1 V, V_C_ = 0.6 V and V_AC_ = 0.6 V, the sensor was insensitive to MF. The sensor became more sensitive to MF at V_B_ = 1.25 V, V_C_ = 2.04 V and V_AC_ =2.04 V, and its output voltage changed from −43.7 mV at −220 mT to 38.6 mV at 220 mT. When V_B_ = 1.5 V, V_C_ = 3.38 V and V_AC_ = 3.38 V, the sensor output voltage obviously increased under different MF. When V_B_ = 1.75 V, V_C_ = 4.58 V and V_AC_ = 4.58 V, the sensor output voltage varied from −120 mV at −220 mT to 119 mV at 220 mT, and the method of least squares was adopted to evaluate the linear regression of the curve. The evaluation obtained that the regression line had a slope of 0.55 V/T and a standard deviation of 5.4 mV. Thereby, the MMF sensor sensitivity in the y-direction MF was 0.55 V/T at bias voltage V_B_ = 1.75 V, V_C_ = 4.58 V and V_AC_ = 4.58 V. 

**Figure 10:**
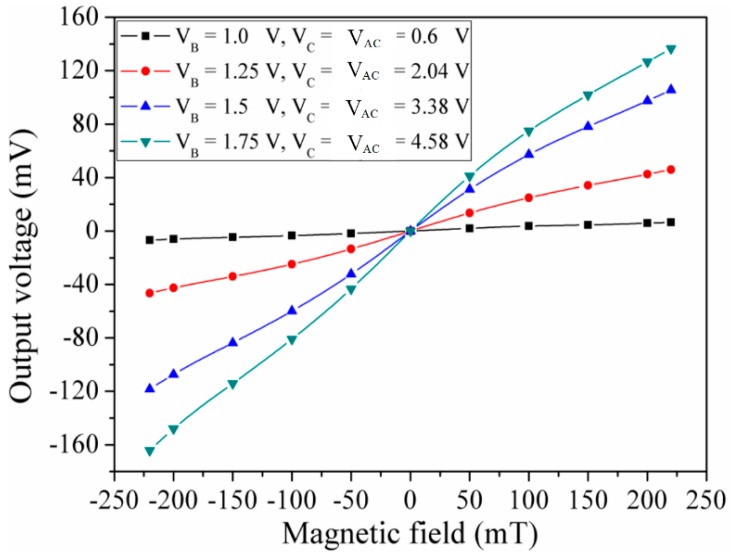
Measured output voltage in the x-direction MF.

With the same testing approach, the sensing performance of the MMF sensor in the x-direction MF was measured. A MF in the x-direction was applied to The MMF sensor. The digital multimeter recorded the voltage difference of the additional collectors AC_2_/AC_4_ in the MMF sensor. Figure 10 demonstrates the measured output voltage of the MMF sensor in the x-direction MF. The sensor was insensitive to MF at V_B_ = 1 V, V_C_ = 0.6 V and V_AC_ = 0.6 V. The sensor was more sensitive to MF at V_B_ = 1.25 V, V_C_ = 2.04 V and V_AC_ = 2.04 V, and it output voltage varied from −46.8 mV at −220 mT to 39 mV at 220 mT. When V_B_ = 1.5 V, V_C_ = 3.38 V and V_AC_ = 3.38 V, the output voltage enlarged under different MF. When V_B_ = 1.75 V, V_C_ = 4.58 V and V_AC_ = 4.58 V, the output voltage changed from −162 mV at −220 mT to 140 mV at 220 mT, and the method of least squares was used to calculate the linear regression of the curve. The calculation obtained that the regression line had a slope of 0.69 V/T and a standard deviation of 12 mV. Thereby, the MMF sensor sensitivity in the x-direction MF was 0.69 V/T at bias voltage V_B_ = 1.75 V, V_C_ = 4.58 V and V_AC_ = 4.58 V. 

**Figure 11:**
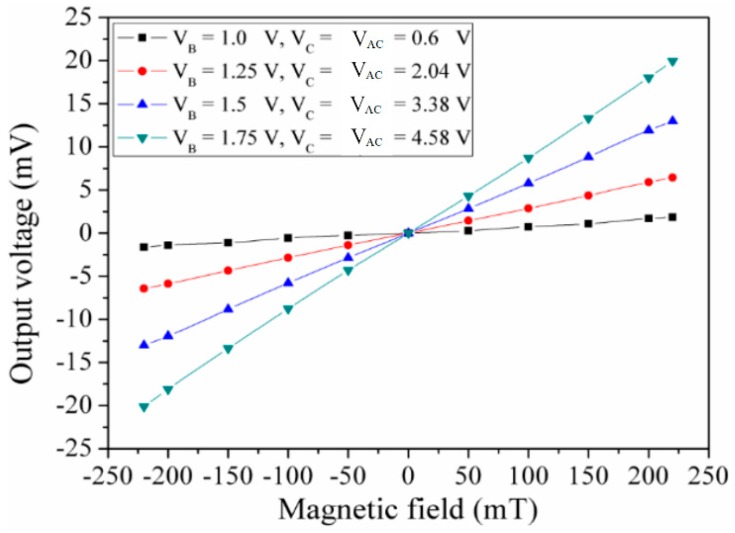
Measured output voltage in the z-direction MF.

The sensing performance of the MMF sensor in the z-direction MF was tested. An MF in the z-direction was applied to the MMF sensor. The digital multimeter measured the output voltage of the Hall electrodes in the MMF sensor. The Hall voltage of the MMF sensor in the z-direction MF was recorded. Figure 11 presents the measured output voltage of the MMF sensor in the z-direction MF. The MMF sensor was also insensitive to MF at V_B_ = 1 V, V_C_ = 0.6 V and V_AC_ = 0.6 V. When bias voltage V_B_, V and V_AC_ increased, the sensor output voltage became large. When V_B_ = 1.75 V, V_C_ = 4.58 V and V_AC_ = 4.58 V, the MMF sensor output voltage varied from −20.5 mV at −220 mT to 20 mV at 220 mT, the curve slope of which was about 0.09 V/T. Thereby, the MMF sensor sensitivity in the z-direction MF was 0.09 V/T at bias voltage V_B_ = 1.75 V, V_C_ = 4.58 V and V_AC_ = 4.58 V.

The characteristics of the MMF sensor in the x- and y-direction MF should be the same owing to the MMF sensor being a symmetric structure. Actually, as shown in Figure 9, the measured sensitivity of the MMF sensor in the y-direction MF was 0.55 V/T. There is little difference in the sensitivity of the MMF sensor in the x- and y-direction MF. The reason is due to packaging and fabrication deviation. As shown in Figure 11, the curves were linear, because the sensing mechanism of the MMF sensor in the z-axis MF was based on the Hall elements. The Hall voltage, which was the output voltage of the sensor in the z-axis MF, was proportional to the magnetic field according to the sensing principle of the Hall element [20]. On the other hand, the sensing mechanism of the MMF sensor in the x- and y-axis MF was based on the magnetotransistor. The carrier current density (Figure 3) of the magnetotransistor depended on the magnetic field, and the I-V (current-voltage) characteristic of magnetotransistor was nonlinear [15], so that the output voltage versus magnetic field (Figure 9 and Figure 10) of the MMF sensor in the x- and y-axis MF was nonlinear.

Table 1 shows a list of sensitivity for various MMF sensors fabricated by CMOS technology. The MMF sensors presented by Tseng [15], Xu [20] and Zhao [21] were one-axis MF sensing, and the sensors proposed by Yu [17], Sung [18] and Yang [22] were two-axis MF sensing. As depicted in Table 1, the sensitivity of the MMF sensor in this work along the x- and y-axis MF exceeds that of Tseng [16], Yu [17], Sung [18] and Yang [22]. The sensitivity of the sensor presented by Zhao [21] along the z-axis MF is higher than that of this work.

## 5. Conclusions

Micro magnetic field sensors were developed employing the CMOS-MEMS technique. The MMF sensors included a magnetotransistor and four Hall elements, where the magnetotransistor could sense MF in the x-axis and y-axis and four Hall elements could detect MF in the z-axis. Additional collectors were added to the magnetotransistor, so that bias current increased and the emitter induced more electron carriers, resulting in enhancing the sensor sensitivity. Manufacturing of the MMF sensors was simple as they did not require post-CMOS processing. Experiments showed that the MMF sensor sensitivity was 0.69 V/T in the x-direction MF at bias voltage V_B_ = 1.75 V, V_C_ = 4.58 V and V_AC_ = 4.58 V, and its sensitivity was 0.55 V/T in the y-direction MF at bias voltage V_B_ = 1.75 V, V_C_ = 4.58 V and V_AC_ = 4.58 V. The sensitivity was 0.09 V/T in the z-direction MF at bias voltage V_B_ = 1.75 V, V_C_ = 4.58 V and V_AC_ = 4.58 V.

## Figures and Tables

**Table 1:** Sensitivity of various MMF sensors.

MMF Sensors	Sensitivity (mV/T)			
	x-axis	y-axis	z-axis
Tseng [15]	354	-	-
Tseng [16]	6.5	6.5	0.4
Yu [17]	38.5	38.5	-
Sung [18]	38.4	38.4	-
Xu [20]	-	-	31
Zhao [21]	-	-	264
Yang [22]	366	365	-
This work	690	550	90
